# Genome-Wide Identification and Comprehensive Analyses of the Kinomes in Four Pathogenic Microsporidia Species

**DOI:** 10.1371/journal.pone.0115890

**Published:** 2014-12-30

**Authors:** Zhi Li, Youjin Hao, Linling Wang, Heng Xiang, Zeyang Zhou

**Affiliations:** 1 College of Life Sciences, Chongqing Normal University, Chongqing, China; 2 The State Key Laboratory of Silkworm Genome Biology, Southwest University, Chongqing, China; 3 College of Animal Science and Technology, Southwest University, Chongqing, China; Institute of Plant Physiology and Ecology, China

## Abstract

Microsporidia have attracted considerable attention because they infect a wide range of hosts, from invertebrates to vertebrates, and cause serious human diseases and major economic losses in the livestock industry. There are no prospective drugs to counteract this pathogen. Eukaryotic protein kinases (ePKs) play a central role in regulating many essential cellular processes and are therefore potential drug targets. In this study, a comprehensive summary and comparative analysis of the protein kinases in four microsporidia–*Enterocytozoon bieneusi*, *Encephalitozoon cuniculi*, *Nosema bombycis* and *Nosema ceranae*–was performed. The results show that there are 34 ePKs and 4 atypical protein kinases (aPKs) in *E. bieneusi*, 29 ePKs and 6 aPKs in *E. cuniculi*, 41 ePKs and 5 aPKs in *N. bombycis*, and 27 ePKs and 4 aPKs in *N. ceranae*. These data support the previous conclusion that the microsporidian kinome is the smallest eukaryotic kinome. Microsporidian kinomes contain only serine-threonine kinases and do not contain receptor-like and tyrosine kinases. Many of the kinases related to nutrient and energy signaling and the stress response have been lost in microsporidian kinomes. However, cell cycle-, development- and growth-related kinases, which are important to parasites, are well conserved. This reduction of the microsporidian kinome is in good agreement with genome compaction, but kinome density is negatively correlated with proteome size. Furthermore, the protein kinases in each microsporidian genome are under strong purifying selection pressure. No remarkable differences in kinase family classification, domain features, gain and/or loss, and selective pressure were observed in these four species. Although microsporidia adapt to different host types, the coevolution of microsporidia and their hosts was not clearly reflected in the protein kinases. Overall, this study enriches and updates the microsporidian protein kinase database and may provide valuable information and candidate targets for the design of treatments for pathogenic diseases.

## Introduction

Microsporidiosis is an opportunistic intestinal infection that is caused by microsporidia, leading to severe human health problems and economic losses. To date, more than 160 genera and 1,300 species of microsporidia are known to cause deadly human diseases and severely damage the silk and beekeeping industries in Asia, North America and Europe [1]–[4]. Unfortunately, there are still no prospective vaccines or drugs to treat microsporidiosis. An unusual group of spore-forming fungi, and the earliest-diverging clade of sequenced fungi [5], microsporidia lack mitochondria or mitochondrial remnants [6]–[8] and are obligate intracellular eukaryotic unicellular parasites. These organisms can suddenly extrude their polar tubes, penetrating the host plasma membrane, and transfer their sporoplasm into host cells, where the spores develop and complete their life cycles [9], [10].

Protein kinases (PKs) are a large group of enzymes that transfer a phosphate group from adenosine triphosphate (ATP) to a number of proteins. Protein phosphorylation functions as a molecular switch for many cellular processes, including metabolism, signal transduction, cell cycle progression, growth and development, and responses to environmental stimuli [11], [12]. Therefore, PKs are considered to be prospective targets for pathogenic disease control. In general, PKs can be categorized into eukaryotic protein kinases (ePKs) and atypical protein kinases (aPKs). Eukaryotic protein kinomes are primarily composed of serine/threonine and tyrosine protein kinases. They share a conserved catalytic domain of 250–300 amino acid residues [13], and contain 12 highly conserved motifs. According to the Hanks and Hunter classification schemes, ePKs are classified into 8 subfamilies, including AGC, CAMK, CMGC, CK1, STE, RGC, TK, TKL, and “Other group” [14]. The atypical protein kinases are classified into small families in virtually all organisms and lack sequence similarity to the typical ePK domain. However, aPKs exhibit protein kinase activity and are homologs of the demonstrated protein kinase [15].

Genomic kinomes have been widely characterized in several organisms from yeasts to humans [16]–[18] and provide an extensive set of probes for investigating the kinases of other species’ genomes. One previous study showed that the pathogenic microsporidium *Encephalitozoon cuniculi* has the smallest eukaryotic genome (2.9 MB) and kinome (29 ePKs and 3 aPKs), with 28% of its protein kinases having no identifiable homologs in model eukaryotes [19]. Their kinomes lack MAP kinase cascades as well as virtually all of the protein kinases involved in the stress response, ion homeostasis and nutrient signaling. In addition to *E. cuniculi*, another microsporidium, *Nosema ceranae*, was identified and sequenced (with a genome size of 7.86 MB) in 2009. Recently, a draft genome (15.7 MB) of the insect microsporidium pathogen *Nosema bombycis* was assembled in our lab [20]. Interestingly, in contrast to *E. cuniculi*, the *N. bombycis* genome has undergone expansion via gene duplication, horizontal gene transfer, and transposable element expansion [20]. Moreover, because *E. cuniculi* and *N. bombycis* live in mammal and insect hosts, respectively, and experience different types of immune resistance, we were interested to know whether this difference in environmental pressures is reflected in their protein kinases.

Because protein kinases play a crucial role in pathogen signal transduction and microsporidia evolution, a genome-wide identification and evolutionary analysis of protein kinases could provide valuable insights into the adaptive diversification of pathogens as well as clues for microsporidia control. In this updated study, we are not only concerning to the basic characterization of kinases in all sequenced microsporidia not just the *E. cuniculi*, but also addressing kinome differences between four microsporidia species, microsporida and model organisms including the gene family expansion and contraction, and phylogeny and selective pressure, the relevance between genome and kinome. Our results showed that although microsporidia adapt to different host types and their genomes present dissimilar evolutionary patterns with regard to expansion and compaction, their kinome appear to be relatively conserved, the coevolution of microsporidia and their hosts was not clearly reflected in the protein kinases.

## Materials and Methods

### Data retrieval

The complete genome sequences of *Enterocytozoon bieneusi*-H348 (ABGB01000000), *E. cuniculi*-GB-M1 (AL391737, AL590442-AL590450), *N. bombycis*-CQ1 (ACJZ01000000) and *N. ceranae*-BRL01 (ACOL01000000) were retrieved from GenBank (http://www.ncbi.nlm.nih.gov/genome/?term=microsporidia).

### Domain analysis and identification

The identified proteins were classified into three major subfamilies based on domain architecture and phylogenetic relationships. All of the kinase domain-containing proteins were downloaded from the kinase database (http://kinase.com/kinbase/FastaFiles/) and Pfam (http://pfam.sanger.ac.uk/family/PF00069). Additionally, two protein kinase domain HMM (Hidden Markov Model) profiles were downloaded from the Pfam (http://pfam.sanger.ac.uk/family/PF00069) and Kinomer (http://www.compbio.dundee.ac.uk/kinomer/index.html) databases. Combined kinase domain HMMs were generated using the Hmmbuild and Cat programs implemented in HMMER 3.1 [21]. The ePKs and aPKs in the four species were identified using hmmsearch in the HMMER package.

The kinase domains were predicted by Pfam [22] and SMART [23]. The candidate kinase domain lengths were expected to cover more than 60% of the defined kinase domains [24]. Multiple sequence alignments were performed using MUSCLE [25]. Conserved motifs and amino acid residues were identified via sequence alignment, and motif graphs were plotted according to their positions by WebLogo (http://weblogo.berkeley.edu/logo.cgi) [26]. Any sequence containing the VAIK, HRD and DFG motifs was considered to be a kinase [15], [27]. Otherwise, it was considered to be a pseudogene. Signal peptides and transmembrane regions were predicted using SignalP4.0 (http://www.cbs.dtu.dk/services/SignalP) and TMHMM (http://www.cbs.dtu.dk/services/TMHMM), respectively. Protein subcellular localizations were predicted by WoLF PSORT (http://wolfpsort.seq.cbrc.jp/) and TargetP (http://www.cbs.dtu.dk/services/TargetP/). Protein kinase functions were predicted by an online GO annotation search (http://bioinformatica.vil.usal.es/lab_resources/pogo/) [28].

### Phylogenetic and evolutionary analysis

A multiple sequence alignment was performed and a phylogenetic tree was generated using PAUP version 4.0b [29] using a neighbor-joining method. The reliability of the tree was tested using bootstrapping with 1000 replicates. Aminoglycoside kinase [APH(3′)III] from the bacterium *Staphylococcus* (P00554) [30], a distant relative of ePK, was used as an outgroup.

To test for the presence of positive selection on the ePKs, only orthologous kinases were selected. The non-synonymous (Ka) and synonymous (Ks) substitution rates were calculated using DnaSP v5.0 [31].

## Results and Discussion

### Genome-wide identification of the protein kinases

**Figure 1:**
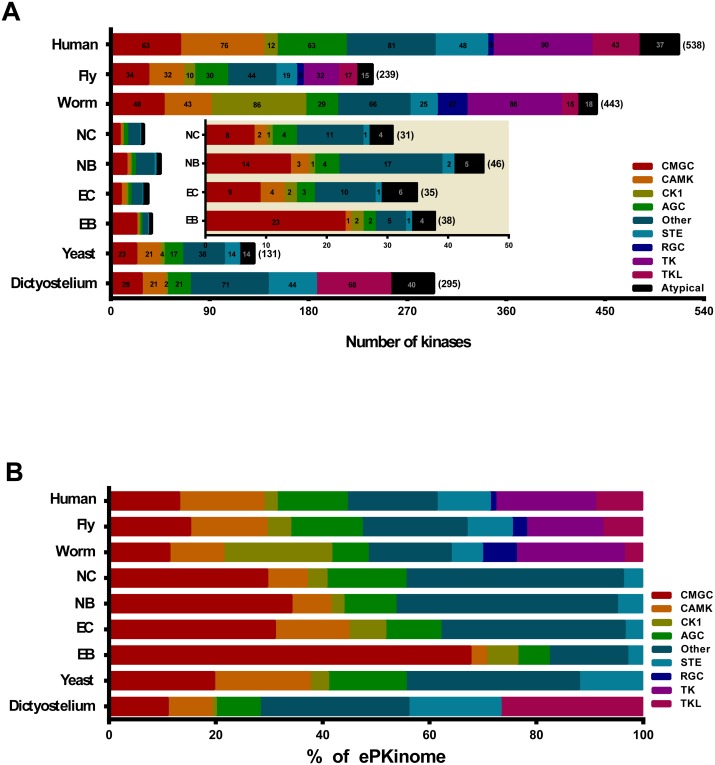
Composition of the Kinomes by Group Level. The kinome compositions are colored by group level. For comparison, the data from the model organisms *Homo sapiens, Drosophila melanogaster, Caenorhabditis elegans, Saccharomyces cerevisiae,* and *Dictyostelium discoideum* are shown in the figure. **A:** the number of protein kinases and the group distribution. The number of protein kinases within each group is numbered with Arabic numerals. **B:** the percentage of the kinome accounted for by the group. The microsporida are indicated as follows: EB, *Enterocytozoon bieneusi*; EC, *Encephalitozoon cuniculi*; NB, *Nosema bombycis*; NC, *Nosema ceranae*.

Genome-wide searches were carried out to classify the protein kinases in the microsporidia species. We identified 34 ePKs and 4 aPKs in *E. bieneusi*, 29 ePKs and 6 aPKs in *E. cuniculi*, 41 ePKs and 5 aPKs in *N. bombycis*, and 27 ePKs and 4 aPKs in *N. ceranae* (Table 1, Fig. 1A). Interestingly, duplication events occurred in *E. bieneusi, E. cuniculi* and *N. bombycis* but not in *N. ceranae* (S1 Table). Compared with a previous report [19], three novel atypical protein kinases, including 2 PIKK and 1 RIO in E. cuniculi, were identified (S1 Table).

**Table 1:** Classification of the Microsporidian Protein Kinases.

Group	Family	Subfamily	*E. bieneusi*	*E. cuniculi*	*N. bombycis*	*N. ceranae*
**AGC**	DMPK	GEK	1	1	2	1
						
	PKA			1	1	1
	***RSK***	***p70***		***1***	***1***	***1***
	PKC	PKC-Alpha	1		1	
	PKC	PKC-Unclassified				1
		**Total**	**2**	**3**	**5**	**4**
**CAMK**	***CAMKL***	***Kin1***	***1***	***1***	***1***	***1***
	***CAMKL***	***CHK1***		***1***	***1***	***1***
	CAMKL	NuaK		1	1	
		**Total**	**1**	**3**	**3**	**2**
**CMGC**	CK2		17	1	1	1
	***CDK***	***CRK7***	***1***	***1***	***3***	***2***
	***CDK***	***CDC2***	***1***	***1***	***3***	***1***
	***CDK***	***CDK7***	***3***	***1***	***3***	***1***
	***CDK***	***PHO85***	***1***	***1***	***3***	***1***
	***CDK***	***CDK9***		***1***		
	***CDK***	***CDK10***		***1***		
	GSK			1	1	1
	DYRK	YAK		1		
	DYRK	DYRK2				1
		**Total**	**23**	**9**	**14**	**8**
**Other**	***CDC7***		***1***	***2***	***2***	***1***
	***Aur***		***1***	***1***	***1***	***2***
	***PLK***		***2***	***2***	***4***	***2***
	WNK		1	1	1	1
	***Wee***			***1***	***2***	***1***
	Haspin			1	2	1
	***TTK***			***1***	***1***	***1***
	NEK				2	1
	PEK	PEK			1	1
	Bud32			1		
	ULK			1		
		**Total**	**5**	**11**	**16**	**11**
**CK1**	CK1		2	2	1	1
		**Total**	**2**	**2**	**1**	**1**
**STE**	STE20	MKC	1	1	2	1
		**Total**	**1**	**1**	**2**	**1**
		**Total ePK**	**34**	**29**	**41**	**27**
**Atypical**	PIKK	ATM	1	1	2	1
	PIKK	ATR	1	3	2	1
	RIO	RIO1	1	1		1
	RIO	RIO2		1	1	1
	RIO	RIO3	1			
		**Total aPK**	**4**	**6**	**5**	**4**

150 kinases were classified into groups, families and subfamilies. The cell cycle-related families are labeled in bold italic typeface.

The predicted protein localizations of the 131 ePKs vary from the cytoplasmic membrane (21%), cytoplasm (26%), nuclear regions (56%) and mitochondria (11%) (S1 Table). None of the microsporidian ePKs contain signal peptides, and 6 of the ePKs possess transmembrane regions (S1 Table).

### Kinome size and density

**Figure 2:**
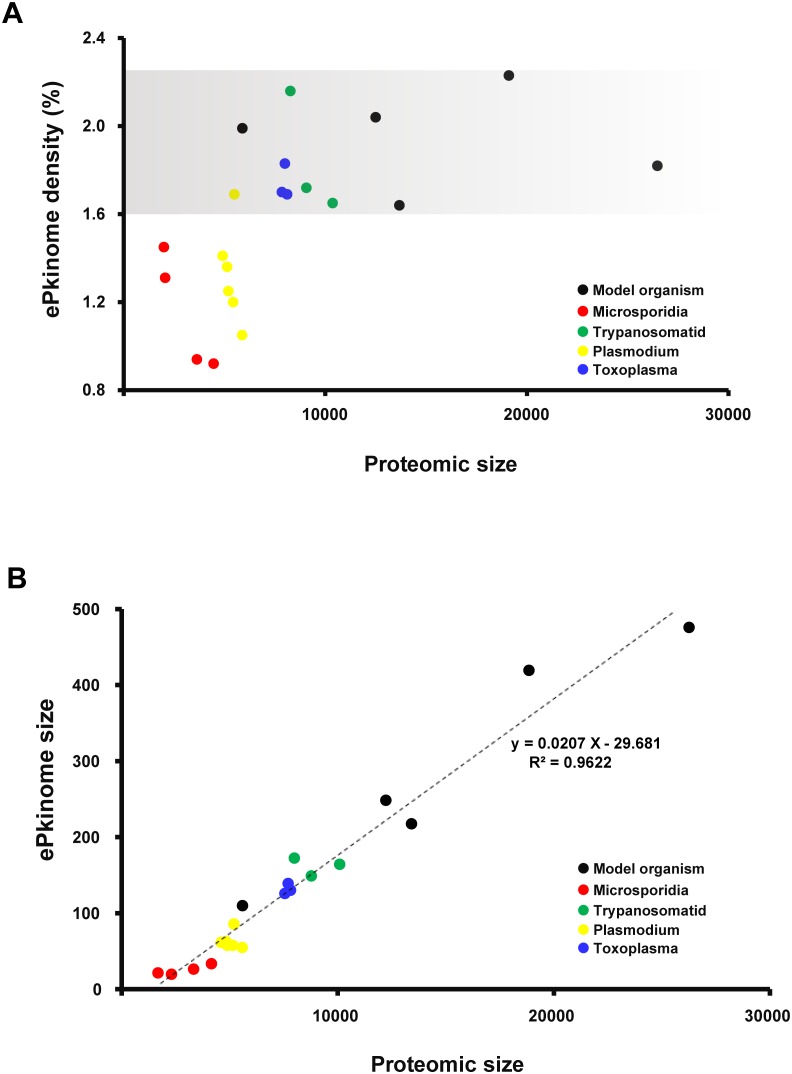
Correlation between Kinome Size, Density and Proteome Size. The ePKinome represents the total number of ePKs in the kinome. Data from the model organisms, the microsporidia, Trypanosomatid, Plasmodium and Toxoplasma were used in the analysis. The scatter plot is colored according to species. The model organism *Homo sapiens*, *Drosophila melanogaster*, *Caenorhabditis elegans*, *Saccharomyces cerevisiae*, *Dictyostelium discoideum* are respective showed as a black scatter plot.

A diverse kinome may provide a more flexible signaling network and help pathogens respond to environmental stimuli, allowing them to survive and infect their hosts. Kinome size is generally related to proteome size in most organisms. Kinome size and density may to some extent reflect the importance of protein phosphorylation in metabolic processes and signal transduction regulation. Parasites appear to be extremely “simple” because they have lost certain organelles and directly access nutrients and energy from their hosts. This unique “simple” condition of parasitic organisms generally results in a large number of lost genes and genome reduction. However, is this simplification reflected on the kinome level? In this study, a total of 27 ePKs in *N. ceranae*, 29 ePKs in *E. cuniculi*, 34 ePKs in *E. bieneusi* and 41 ePKs in *N. bombycis* were identified (Fig. 2A). The number of ePKs (27 kinases) in *N. ceranae* is smaller than the minimal kinome of the human intestinal pathogen *Giardia lamblia* (80 kinases) [32] and is the smallest of the kinomes studied. This finding suggests that microsporidian kinomes are the smallest eukaryotic kinomes characterized to date. In addition, our data show that microsporidian kinome density ranges from 0.92% to 1.45%, which is also remarkably smaller than the kinome density ranges of model organisms (1.6–2.2%) (Fig. 2A). Recently, striking data showed that although the proteome size is highly variable in the fungal family Ascomycetes, their kinome sizes appear to be stable [33]. However, quite unlike Ascomycetes, the microsporidian kinome sizes appeared to be reduced, albeit with increased kinome densities accompanying proteome compaction (Fig. 2A). In addition, a significant linear positive correlation between kinome and proteome size was discovered in the present study (Fig. 2B). The number of kinases increases with kinome size expansion in several organisms, including Trypanosomatid, Plasmodium, Toxoplasma and the model organisms (Human, Worm, Fly, Yeast) considered in this study (Fig. 2B). Moreover, although there is no remarkable correlation between the kinome density and proteome size in the organisms at the phylum level (Fig. 2A), kinome density increased with proteome size reduction within the same genus (Fig. 2A). These data suggested the higher degree of species differentiation, the more complex correlation between kinome density and proteome size. It may to some extent reflect the importance of protein phosphorylation in organisms.

### Domain composition and conserved features

**Figure 3:**
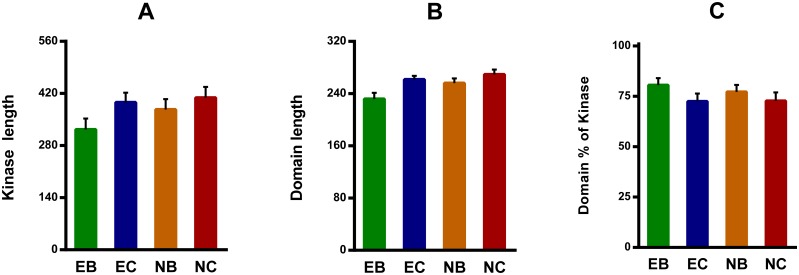
Comparison of ePK Sequence Lengths in the Microsporidia. A, B and C show a comparison of the sequence lengths of the domains, the sequence lengths of the full proteins, and the percentage of the domain overlaps with the full proteins, respectively. The microsporidia are abbreviated as follows: EB, *Enterocytozoon bieneusi*; EC, *Encephalitozoon cuniculi*; NB, *Nosema bombycis*; NC, *Nosema ceranae*.

**Figure 4:**
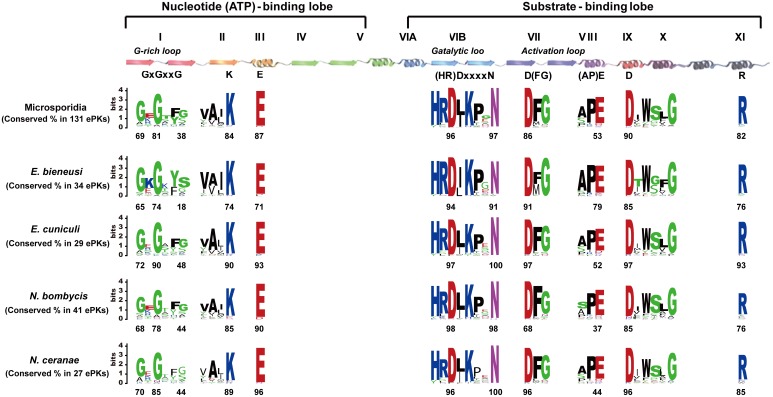
Conservation of ePK Domains, Key Motifs, and Residues. The overall height of a stack indicates the sequence conservation at that position. The height of the symbols within each stack indicates the relative frequency of the key amino acid at that position. The conservation of key residues is presented as a percentage and labeled at the bottom of each logo.

In general, ePKs are characterized by the presence of a highly conserved catalytic domain with 250–300 amino acid residues divided into 12 subdomains. The N-terminal subdomains I–IV participate in nucleotide binding, whereas the C-terminal subdomains VIA-XI are involved in phosphotransfer and protein-substrate-binding. Subdomain V serves as an intervening linker [34]. In this study, 123 ePKs contained only one functional catalytic domain, 2 ePKs had a dual kinase catalytic domain, and 6 ePKs contained one accessory domain (S1 Table). The microsporidian ePKs catalytic domain consists of 230–270 amino acid residues and accounts for 73–81% of its full length (Fig. 3). In the four microsporidia, no significant difference was present in the sequence length of the domain or full protein, and the percentage of the domain overlaps with the full protein (Fig. 3). Overall, microsporidian ePKs catalytic domains, critical amino acid residues and motifs are relatively conserved, even if some of the ePKs are less conserved than the kinases in the model organisms investigated in this study (Fig. 4 and S1 Fig.).

Ordinarily, eukaryote protein kinase subdomain I has a glycine-rich loop for ATP binding (G-X-G-X-X-G). However, this loop appears to be less conserved in microsporidia, especially in the third glycine residue (G), which has only 18–48% conservation (Fig. 4). This result suggests that microsporidian ePKs cannot form a stable β-strand-turn-β-strand structure to cover and anchor the non-transferable phosphate groups of ATP. The glutamate residues (E) in subdomain III are typically located in the helix and form salt bridges to stabilize the interactions between lysine and the α- and β-phosphates of ATP [15]. Like the majority of ePKs, microsporidian glutamate residues are well conserved in subdomain III. In addition, subdomain VIII has a highly conserved PE motif, in which the E residue plays an important role in the recognition of peptide or protein substrates. Surprisingly, the glutamate in subdomain VIII is not highly conserved (with an average of 53%) in the microsporidian kinases, notably in *N. bombycis* (only 37%), which is rare for most eukaryotes. This result implies that the microsporidian ePKs could not directly recognize or bind their substrates during protein phosphorylation. It is also probable that mutations in key amino acid residues affect protein structure, which facilitates substrate binding and contributes to the adaptability of microsporidian parasites.

The invariant VAIK, HRD and DFG residues in subdomains II, VI and VII are required for protein kinase phosphorylation. If a protein lacks any of these key residues, it is considered to be an inactive kinase [15], [27]. The lysine residue (K) contacts the α- and β-phosphates of ATP, primarily participating in anchoring and orienting ATP. The HRDxxxxN motif plays a role in the transfer of a phosphate group from ATP to an appropriate substrate. Protein kinases with DFG motifs can bind the Mg2+ interacting with the β-and γ-phosphates of ATP. Following these criteria, although catalytically essential residues (VAIK, HRD and DFG) or motifs were conserved well in the four microsporidia, there are 12 ePKs in *E. bieneusi*, 3 ePKs in *E. cuniculi*, 7 ePKs in *N. bombycis*, and 3 ePKs in *N. ceranae* still predicted to lack catalytic activity. The number of ePK pseudogenes in the microsporidia (10–35%) appears to be much higher than that in the human kinome (∼10%) [27]. Some of these pseudogenes lack not only the residue itself but also the entire conserved motif (S1 Table). Therefore, further experiments will be needed to confirm this prediction.

The domain compositions of microsporidian ePKs appear to be simple; only one accessory domain was identified in the GEK kinase present in all of the microsporidia, C1_1 (CL0006), and a Polo box domain was present in *E. cuniculi* and *N. bombycis* PLK kinases. An HR1 repeat is distributed in the p70 kinase of *E. cuniculi* (S1 Table). The microsporidian kinome has far fewer accessory domains than the human kinome, where over 50% of the kinases contain additional domains [27]. Thus, the microsporidia genome appears to be highly reduced and compact when compared with other species. The loss of gene segments and/or domains may explain why microsporidian protein kinases contain minimal accessory domains.

### Classification of microsporidian protein kinases

**Figure 5:**
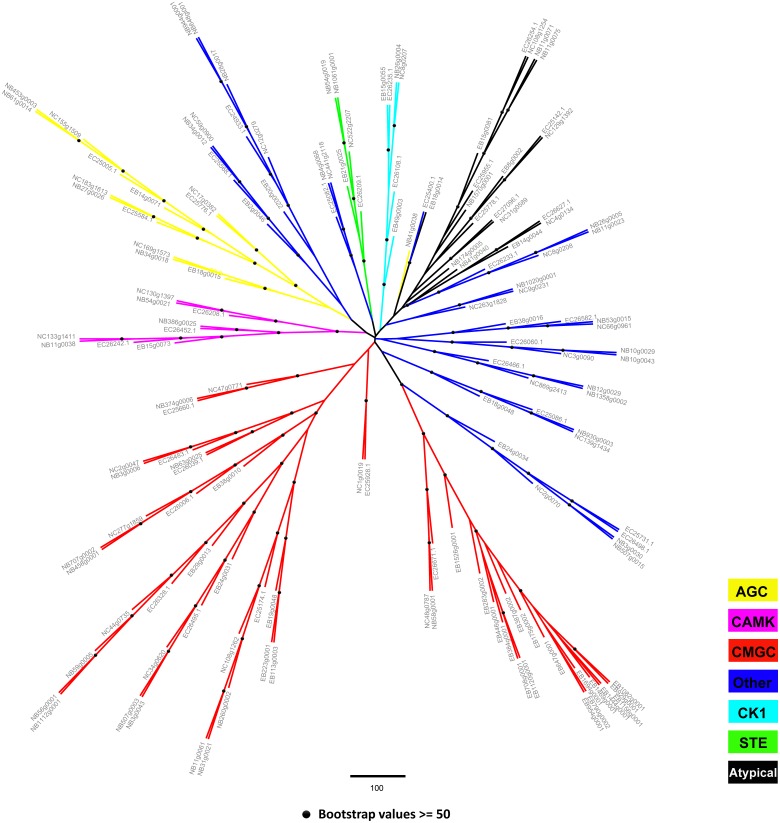
Unrooted Tree Representation of the Microsporidian Kinome. An unrooted tree was constructed using the catalytic domain sequences. The classification of the protein kinase is colored by group level. The species names are abbreviated as follows: EB, *Enterocytozoon bieneusi*; EC, *Encephalitozoon cuniculi*; NB, *Nosema bombycis*; NC, *Nosema ceranae*.

To gain further insight into the different classes of kinases, a combined approach based on sequence similarity and phylogenetic relationships in conserved catalytic domains was used. In total, 150 protein kinases were classified into the AGC, CAMK, CMGC, CK1, STE, Other, and Atypical groups (Fig. 5, Table 1). Among these, the predominant groups were CMGC and Other, and the relatively smaller groups were CK1 and STE. The RGC, TK and TKL groups were not present in the microsporidia. Detailed information regarding these groups is summarized in Table 1 and S1 Table and S2–S7 Figs.

#### The AGC group

There were 2 ePKs in *E. bieneusi*, 3 ePKs in *E. cuniculi*, 5 ePKs in *N. bombycis*, and 4 ePKs in *N. ceranae* that were identified as members of the AGC group (S2 Fig.). GEK (Genghis Khan) kinase, an AGC kinase group member, belongs to the myotonic dystrophy protein kinase (DMPK) family that regulates cell size and shape in a variety of organisms by interacting with members of the Rho family of small GTPases [35]. The DMPK family was also distributed in each microsporidian kinome, suggesting that DMPK is well conserved and may also play an important role in microsporidian signal transduction. Protein kinase C-alpha (PKC-α), also in the AGC group, was generally activated by calcium and the second messenger diacylglycerol and was previously found to be involved in host innate immune responses to the parasite *Toxoplasma gondii* [36], [37]. In yeast, PKC-α can stimulate calcium uptake and regulate cell wall metabolism via the MAP kinase cascade [38]. Here, PKC-α homologs were also identified in *E. bieneusi, N. bombycis* and *N. ceranae*, but their roles in the microsporidian parasite life cycle remain unknown. Protein Kinase A (PKA), a cAMP-dependent protein kinase, is conserved in eukaryotes and is activated by cAMP production downstream of G protein coupled receptors. Previous researchers have shown that *Saccharomyces cerevisiae* PKA is required for spore germination in response to glucose [39], [40]. However, *S. cerevisiae* PKA can also regulate gametocytogenesis in *Plasmodium falciparum* as well as morphogenesis in Dictyostelium [41], [42]. In the present study, PKA was identified in all of the microsporidian kinomes with the exception of *E. bieneusi*. Our data suggest that PKA may be involved in the invasion processes of microsporidia. In addition, three ribosomal s6 kinase (RSK) families are absent from the *E. bieneusi* kinome. RSK kinases are important regulators of protein synthesis during endothelial cell proliferation [43]. All of the RSKs lack the key VAIK, HRD and DFG residues in their corresponding subdomains, which suggests that they are catalytically inactive in microsporidia (S1 Table).

#### The CAMK group

Members of the CAMK (calcium/calmodulin-regulated kinase) group play a crucial role in signal transduction and are associated with protein secretion, transport and other biological processes. We found 1 such kinase in *E. bieneusi*, 2 in *N. ceranae*, 3 in *E. cuniculi* and 3 in *N. bombycis* (Table 1, S3 Fig.). These kinases are CAMK-like family members, including Kin1, Chk1 and the Nuak subfamily. It is worth noting that the protein EC26060.1 in *E. cuniculi*, which was previously identified as a member of the CAMK family [19], was clustered into the Other group based on phylogenetic analysis and a comprehensive comparison (S5 Fig.).

In the pathogen *Cryptococcus neoformans*, Kin1 plays an essential role in escaping the host’s immune recognition system, and Kin1 mutations can increase binding to alveolar and peritoneal macrophages [44]. In *Ustilago maydis*, Kin1 is associated with polar hyphal growth and vacuolar organization [45], [46]. In *Schizosaccharomyces pombe*, Kin1 is important for cell polarity and cytoskeletal dynamics [47]. In *S. cerevisiae*, Kin1 mutations can cause morphology and growth defects [48]. Kin1 was present in all the microsporidian genomes in this study and is likely to be involved in some of the functions noted above.

Chk1, a mitogen-activated protein kinase, is considered to be the most important cell cycle regulatory kinase in eukaryotic organisms [49]. In the phytopathogenic fungus *U. maydis*, Chk1 is involved in the DNA-damage response during cell cycle arrest [50]. Moreover, in the corn pathogen *Cochliobolus heterostrophus*, Chk1-deleted mutants can reduce infection rates by delaying the penetration of hyphae into leaves [51]. In *E. cuniculi*, Chk1 putatively plays crucial roles in replication and/or DNA damage repair. Thus, we speculated that microsporidian Chk1 may play similar roles in spore development, growth and/or the cell cycle. The Chk1 that was lost from the *E. bieneusi* genome may have been replaced by another gene with a function similar to that of Chk1.

Chk1 and Rad53 control cell cycle progression and DNA damage checkpoints by regulating downstream gene expression [52]–[54]. The kinase EC25086.1 was previously identified as a member of the CAMK group but did not fall into any defined subfamily in the *E. cuniculi* genome [19]. In this study, EC25086.1 was clustered into “Other group,” rather than the Rad53 family, after a combined BLASTP and phylogenetic analysis (S5 Fig.). The loss of Rad53 in the microsporidian genome suggests that other kinases may be able to undertake its functions.

In addition, CDPKs (calcium-dependent protein kinases) are widely exist in apicomplexan parasites, ciliates, green algae, and plants. They are involve in regulating calcium signals, have a variety of functions in transcription, metabolism, ion pumps and channels, and the cytoskeleton [55]–[57]. These superfamily kinases are particular important in malaria parasite life cycle. However, no CDPK or CDPK-like kinases were identified in the microsporidian genomes.

#### The CMGC group

The CMGC group is the most prominent group in the microsporidian kinome, but the number of kinases in the CMGC group is less numerous than those in the model organisms studied. CMGC group kinases are often highly conserved across organisms and play an important role in controlling cell proliferation and development. The relative abundance of CMGC in the microsporidian kinome may promote spore proliferation and development during the microsporidian parasitic life cycle. In a previous report, two kinases, EC26498.1 and EC25731.1, were classified into the CDC7 family of the CMGC group [19]. In this study, however, EC26498.1 and EC25731.1 clustered together with other kinases in *E. bieneusi, N. bombycis, N. ceranae* and yeast, forming a clear cluster within the Other group (S4 Fig.).

The cyclin-dependent kinase (CDK) family in the CMGC group is large and essential for the completion of START and controlling the events in the cell cycle required to initiate mitosis [58]. In microsporidia, CDK is a prominent family that includes the CDC2, PHO85, CDK7, CRK7 subfamilies. Previous studies have shown that the cdc2 gene is the rate-limiting factor for the initiation of both the mitotic S-phase and M-phase due to its regulation of DNA synthesis in *S. pombe* [58], [59]. CDC2 kinase was present in all of the microsporidia, suggesting that this kinase is well conserved and essential for microsporidian proliferation and development. Generally, cell proliferation plays a crucial role in a parasite’s life. CDC2 kinase is essential for cell cycle regulation. In *S. cerevisiae*, the Pho85/Pho82 complex is essential for driving cell-cycle progression and nutrient metabolism, such as phosphate and carbon sources [60]. Furthermore, Pho85 plays a role in cellular responses to changes in the extracellular environment [60]. Six Pho85 kinases were identified for the first time in the four microsporidian kinomes, including 3 copies in *N. bombycis*. A sequence analysis indicated that microsporidian Pho85 shares a high similarity with *S. cerevisiae* Pho85. Therefore, we speculate that the Pho85 gene began its genetic differentiation in microsporidia. Taken together, our data suggest that Pho85 plays an important role in the microsporidian cell cycle, and regulates their perception of and response to stress from the environment. CDK7 is the essential component of the cell cycle and transcription initiation factor TFIIH. This kinase functions as a CDK-activating kinase (CAK) and is involved in transcription initiation and DNA repair [61]–[63]. Arabidopsis CRK7 is closely related to cdc2 kinase and is required for RNA splicing and mediates the response to extracellular but not chloroplastic ROS production [64], [65]. In the present study, a novel CRK7 kinase was discovered in the *E. cuniculi* genome. Our data show that all four of the microsporidia contain the CDK7 and CRK7 kinases and that these kinases may play an essential role in microsporidian proliferation.

In addition to the CDK family, the DYRK, GSK and CK2 families were found in the microsporidian kinomes. The DYRK proteins have been widely characterized in *S. cerevisiae* and *S. pombe*. These kinases are related to cell cycle control, cytokinesis, cell differentiation, development and cell homeostasis [66]. In this study, DYRK kinases were found in *E. cuniculi* and *N. ceranae*. This result suggests that the function of DYRK is not required for all microsporidia. Similarly, GSK family members, which were only found in *N. ceranae*, were not conserved in the microsporidia. Previous reports have shown that GSK is involved in embryogenic segmentation and neurogenesis in Drosophila [67], [68], cell fate regulation in Dictyostelium [69] and axis formation during Xenopus development [70], [71]. In addition to its role in development, *S. cerevisiae* GSK kinase activates the Msn2p-dependent transcription of stress responsive genes. It is unknown why this important kinase was lost in *N. ceranae*. Casein kinase 2 (CK2), an extremely well-conserved eukaryotic protein kinase, participates in a wide range of functions. In *S. cerevisiae*, CK2 is a major regulator of G1/S transition via the phosphorylation of Sic1 and Cdc34 [72]–[74]. In addition, CK2 is induced by salt stress and increases NaCl tolerance in *S. cerevisiae* [75]. In the present study, CK2 was conserved in all four species. Surprisingly, *E. bieneusi* has 17 CK2 kinases. It is rare for such a high number of CK2 kinases to be present in one organism. In the phylogenetic tree, *E. bieneusi* CK2 kinases form a separate sister branch and then cluster with other CK2 kinases (S4 Fig.). A sequence alignment showed that *E. bieneusi* CK2 kinases share high sequence identities with the CK2 kinases from model organisms but have incomplete catalytic domains. Among the *E. bieneusi* CK2 kinases, 6 are missing the N-terminal and the other 11 are missing the C-terminal. In addition, 10 of the kinases are not likely to have catalytic activity because they are missing the critical amino acid residue K in subdomain II in addition to residue D in subdomains VI and VII (S1 Table). Taken together, these findings suggest that *E. bieneusi* CK2 may have been produced by a birth-and-death evolutionary process, but further data are needed to confirm this possibility.

Overall, the key kinases involved in the regulation of cell cycle progression, CDK and CK2, are well conserved in the microsporidia. In contrast, the CDK9, CDK10 and YAK kinases are not present in all of the microsporidian genomes, which may be attributed to their coevolution with different hosts. Unfortunately, none of the kinases participating in the MAPK pathway were found in this study. The MAPK cascades have been shown to have important functions in proliferation, differentiation, development, transformation, and apoptosis [76]–[78]; gametogenesis and transmission [79]; osmotic and nutrient stress responses [80], [81]; and parasite invasion [82]. As a group of eukaryotic intracellular parasites, it is unclear why microsporidia do not possess the MAPK signal pathway. Based on our data that MAPK system occurred before microsporidia even not complete (S1 Table), we speculated that MAPK cascades was lost in microsporidia since the fungal split from animals.

#### The Other group

The proportion of the Other group is 15–32% of the kinome of the model species (Fig. 1B), 15% in the helminth parasite *Schistosoma mansoni* [83] and 25% in Trypanosomatids [12]. Surprisingly, the Other group is much larger in the microsporidia than in the representative organisms; for example, it represents 41% of the total number of kinases in *N. bombycis* (Fig. 1B). In addition, the microsporidian Other group members are well conserved and mainly consist of cell cycle-related kinases. In total, forty-four genes are categorized into twelve families within the Other group. Among these genes, more than half of the kinases are grouped into the families CDC7, Aur, PLK and WNK (S5 Fig.). However, the NEK and PEK families only exist in the insect microsporidia *N. bombycis* and *N. ceranae*. WEE, Hspin and TTK have been lost in E. bieneusi (S1 Table). The CDC7 kinase family is conserved in eukaryotes and involved in cell cycle regulation. In *S. cerevisiae*, the CDC7 family protein is required for the G1/S transition during the cell cycle [84], [85]. AUR and PLK family members play an essential role in mitotic spindle formation [86], [87]. The TTK family kinase in *S. cerevisiae* is associated with centrosomal duplication. Fission yeast WEE family proteins are negative regulators of mitosis [88]. Recently, yeast Haspin kinase was shown to be necessary for mitotic spindle positioning and mitotic arrest regulation [89]. In this study, the homologs of this kinase were found to be well conserved in microsporidia. These results suggest that they are essential for the microsporidian cell cycle.

The WNK (with no lysine kinase) family members are also well conserved in the microsporidia. WNK often lacks the key lysine residue in catalytic subdomain II and is involved in cell adhesion and tissue formation [90]. A sequence similarity analysis showed that the WNK proteins (EBI_38g0016, EC26582.1, NBO_53g0015 and NCE_66g0961) indeed lack the conserved amino acid residue K in subdomain II (S1 Table). In the phylogenetic tree, these proteins are clearly clustered with human, fruit fly and nematode WNK family members (S5 Fig.). A number of studies have provided compelling evidence for microsporidian spore adhesion as a critical step in host cell infection [91], [92]. Despite extensive research, however, the fundamental signal transduction mechanisms responsible for spore adhesion and host infection have not yet been fully elucidated. The WNK kinase distributed in microsporidia may play a role in the adhesion of spores to host cells during infection.

#### The CK1 group

CK1 group (including casein kinase 1 and its close relatives) consists of seven family members and is widely distributed in eukaryotic organisms from yeasts to humans. CK1 group members participate in the Wnt signaling pathway by phosphorylating disheveled [93], and participate in mammalian circadian rhythm regulation [94]. In *S. cerevisiae*, CK1 is important for the delivery of proteins to the vacuole after endocytosis [95], and is required for receptor phosphorylation [96]–[98]. In the present study, four CK1 kinases were identified in three microsporidian genomes. Similar to the model organisms, with the exception of *Caenorhabditis elegans*, which possesses the largest CK1 group of all the model organisms, microsporidia contain few CK1 kinases in their genomes (S1 Table). Although BLASTP showed that the EBI_49g0003 protein of *E. bieneusi* shares sequence similarity with yeast STE, evidence from the phylogenetic tree supports it being a member of the CK1 group (S6 Fig.). Although growing evidence suggests that microsporidia evolved within the kingdom Fungi [99], the microsporidian CK1 proteins (EBI_15g0055, EC26235.1, NBO_26g0004, NCE_8g0207,) were not closely clustered with those of the fungus *S. cerevisiae* (S6 Fig.).

#### The STE group

The STE group consists of the members of the STE7, STE11, and STE20 families. These proteins are generally involved in MAPK activation or regulation of the cytoskeleton [100], [101]. Although no MAPK-related kinases were identified in the microsporidia genomes, STE20 kinase homologs were found in each of them (EBI_21g0025, EC26209.1, NBO_54g0019, NBO_1061g0001 and NCE_522g2207) (S1 Table). The number and percentage of STE kinases are still much smaller than those in other model organisms. In the phylogenetic tree, the microsporidian MKC kinases are clustered into a small group (S7 Fig.). Our findings update previous research that did not find STE family kinases in *E. cuniculi* [102].

#### Atypical protein kinases

Atypical protein kinases (aPKs) were first found in the human genome and have low sequence similarities to known ePK domains. However, they are known to possess protein kinase catalytic activity [27]. In this study, fewer than 6 atypical kinases were identified in each microsporidian species, (S1 Table), and these atypical kinases were assigned to the RIO or PIKK (Phosphatidyl inositol 3′ kinase-related kinase) families. In *S. cerevisiae*, RIO kinase plays a crucial role in ribosome biogenesis and cell cycle progression [103]. Loss of the RIO gene can affect an organism’s growth rate [104]. However, the functions of microsporidian RIO and PIKK remain unclear.

### Phylogenetic and evolutionary analysis of the protein kinases in microsporidia

**Figure 6:**
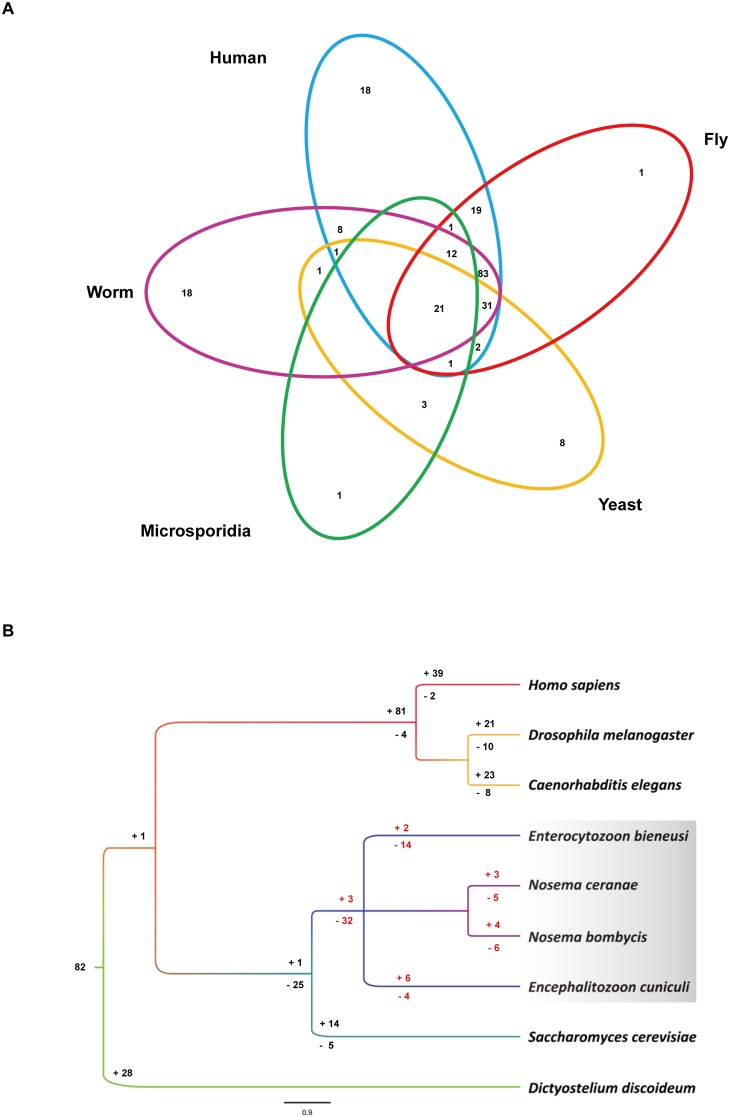
Kinase Subfamily Gain and Loss. A: Subfamily distributions in the microsporidia and model organisms. B: Subfamilies lost in the microsporidia.

A phylogenetic analysis revealed that microsporidia form a sister group of all sequenced fungi, establishing an earliest-diverging clade of fungi [5]. However, the microsporidian kinase subfamily evolutionary pattern is still unclear. Gene gain and/or loss is summarized in Fig. 6 and S1 Table.

The microsporidia, Dictyostelium, yeast, worm, fly and human kinomes contain a common ancestor with 82 distinct subfamilies, but nearly three-quarters of these subfamilies have been lost in microsporidia (Fig. 6). Microsporidia share 35 subfamilies with the fly and human kinomes, 33 with the worm kinome, and 25 with the yeast kinome. Because the microsporidia are unicellular eukaryotic animal pathogens, the coevolution of microsporidia with their hosts may cause a gain or loss of kinases. In addition, microsporidia lack certain kinases that are well conserved in other parasitic pathogens, such as those for nutrient and energy signaling and the stress recognition and response pathway. Their loss of stress-responsive kinases may explain why microsporidia are not sensitive to chemical and/or biological signals from the external environment. They have dense and rigid spore walls containing chitin to help their spores resist various environmental challenges [105], [106]. When yeasts and microsporidia diverged, 25 kinase subfamilies were lost from the microsporidia kinomes. An additional 46, 37, 38 and 36 kinase subfamilies were lost in *E. bieneusi, E. cuniculi, N. bombycis* and *N. ceranae*, respectively (Fig. 6). Thus, gene loss is most apparent in *E. bieneusi*. Overall, a large number of kinases involved in metabolic and signal transduction have been lost in microsporidia. However, many cell cycle-related kinases have been preferentially retained in the kinomes of the microsporidia (Table 1). In contrast, microsporidia have gained a few novel kinase subfamilies compared with *Dictyostelium discoideum*. These additional kinases have multiple functions in the cell cycle and contribute to the parasitic lifecycle of microsporidia. Recently, it was reported that the microsporidium *N. bombycis*’ genome has expanded in comparison with the human parasite *E. cuniculi* [20]. However, our results reveal that *E. cuniculi* lost 4 subfamilies but gained 6 subfamilies, and *N. bombycis* lost 6 subfamilies but gained 4 subfamilies (Fig. 6). Therefore, there were no remarkable differences in gene gain/loss or subfamily size in the four microsporidia, even though their genomes present dissimilar evolutionary patterns with regard to expansion and compaction. Previously, 15 kinase subfamilies were found to be nematode specific [107], 13 subfamilies were found to be specific for humans and 7 subfamilies were found to be specific for fruit flies [16], [107]. *S. cerevisiae* also possesses fungal-specific protein kinase families, including CAMKL in the CAMK group and RAN, HAL and CAMKK in the “Other group” [108]. These kinases generally respond to osmotic stress and regulate the cell cycle. Interestingly, 2 fungal-specific kinase subfamilies were also conserved in the microsporidian kinomes, including PKC-Unique in the AGC group and Kin1 in the CAMK group (S1 Table). Moreover, in contrast with *S. cerevisiae*, the microsporidia gained cell procession-related kinases such as the PHO85 kinase in the CMGC group (Table 1, S1 Table). However, the microsporidia also lost important kinases that may play critical roles in metabolism, apoptosis, cellular proliferation, differentiation, mitotic progression, cytokinesis and morphogenesis [109], [110].

### Substitution rate estimates for the ePKs in microsporidia

**Figure 7:**
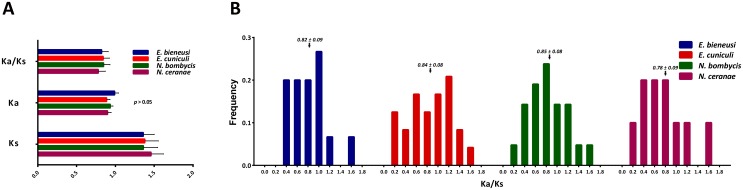
Nonsynonymous (Ka), Synonymous (Ks) and Ka/Ks Ratio Calculations. Gene sequence alignments were generated based on the best matches to the human kinase reference at the subfamily level. The Ka, Ks, and Ka/Ks ratios were analyzed to assess and compare the differences in selection pressure among the four species.

Our previous study revealed that the *N. bombycis* genome has undergone expansion by gene duplication [20]. However, the genome of *E. cuniculi* has been reduced. Thus, Ka/Ks ratios were calculated to discern whether positive selection acts on microsporidian kinomes. The results showed that the Ka, Ks and the distribution of the Ka/Ks ratios are not significantly different in the four microsporidia (Fig. 7). Although the average Ka/Ks ratio in all the microsporidia was less than 1, several kinases within each microsporidian genome had Ka/Ks ratios that were greater than 1. Our results suggest that the protein kinases in each genome are under strong purifying selection pressure and that positive may have acted on only a few sites during the evolutionary process.

## Conclusion

Microsporidian kinomes consist of the AGC, CAMK, CMGC, CK1, STE, Other, and Atypical groups, but lack RGC, TK and TKL groups. Their ePKs catalytic domains, critical amino acid residues and motifs are relatively conserved. Some kinases involved in nutrition and energy signaling, and stress responses, were lost. But genes related to cell cycle, development and growth were well conserved.

Our results revealed the following novel findings: 1), the *N. ceranae* kinome (27 ePKs and 4 aPKs) except *E. cuniculi*, is the smallest eukaryotic kinome characterized to date; 2), microsporidian kinome size reduction is in good agreement with proteome compaction, albeit with increased kinome densities accompanying proteome reduction; 3), microsporidia share 35 subfamilies with the fly and human kinomes, 33 with the worm, and 25 with the yeast. Nearly three-quarters of the kinase subfamilies from a common ancestor were lost in the microsporidia including some critical kinases in metabolism, apoptosis, cellular proliferation, differentiation, mitotic progression, cytokinesis and morphogenesis process; 4), the coevolution of microsporidia and their hosts is not reflected in protein kinases. In both the mammal- and insect-pathogenic microsporidia, no remarkable differences were observed in kinase family distribution, domain architecture, key amino acid residues, gene gain and loss, or selective pressure.

In conclusion, our results further enriched and updated the microsporidian protein kinase database, provide some valuable information for future functional investigations of protein kinase gene family for drugs identify candidates to control pathogenic diseases.

## Supporting Information

S1 Fig **Multiple Sequence Alignment of the ePK Domains.** The domain sequences were aligned without modifications. The kinase names are shaded according to species: green, *Enterocytozoon bieneusi*; purple, *Encephalitozoon cuniculi*; orange, *Nosema bombycis*; red, *Nosema ceranae*. The 12 conserved subdomains are numbered according to the Hanks and Hunter classification system. The positions of the conserved amino acid residues are indicated with Arabic numerals.
(TIF)

S2 Fig **Phylogram of the AGC Kinases.** A phylogenetic tree based on the catalytic domains was constructed as described in the Methods section. Aminoglycoside kinase (APH3) was used as an outgroup. The microsporidian ePKs are labeled in red. The model organism names are abbreviated as follows: Hs, *Homo sapiens*; Dm, *Drosophila melanogaster*; Ce, *Caenorhabditis elegans*; Sc, *Saccharomyces cerevisiae*. The bootstrap values (> = 50) are showed at each node of the phylogenetic trees.
(TIF)

S3 Fig **Phylogram of the CAMK Kinases.** A phylogenetic tree based on the catalytic domains was constructed as described in the Methods section. Aminoglycoside kinase (APH3) was used as an outgroup. The microsporidian ePKs are labeled in red. The model organism names are abbreviated as follows: Hs, *Homo sapiens*; Dm, *Drosophila melanogaster*; Ce, *Caenorhabditis elegans*; Sc, *Saccharomyces cerevisiae*. The bootstrap values (> = 50) are showed at each node of the phylogenetic trees.
(TIF)

S4 Fig **Phylogram of the CMGC Kinases.** A phylogenetic tree based on the catalytic domains was constructed as described in the Methods section. Aminoglycoside kinase (APH3) was used as an outgroup. The microsporidian ePKs are labeled in red. The model organism names are abbreviated in the kinase names as follows: Hs, *Homo sapiens*; Dm, *Drosophila melanogaster*; Ce, *Caenorhabditis elegans*; Sc, *Saccharomyces cerevisiae*. The bootstrap values (> = 50) are showed at each node of the phylogenetic trees.
(TIF)

S5 Fig **Phylogram of the Other Kinases.** A phylogenetic tree based on the catalytic domains was constructed as described in the Methods section. Aminoglycoside kinase (APH3) was used as an outgroup. The microsporidian ePKs are labeled in red. The model organism names are abbreviated in the kinase names as follows: Hs, *Homo sapiens*; Dm, *Drosophila melanogaster*; Ce, *Caenorhabditis elegans*; Sc, *Saccharomyces cerevisiae*. The bootstrap values (> = 50) are showed at each node of the phylogenetic trees.
(TIF)

S6 Fig **Phylogram of the CK1 Kinases.** A phylogenetic tree based on the catalytic domains was constructed as described in the Methods section. Aminoglycoside kinase (APH3) was used as an outgroup. The microsporidian ePKs are labeled in red. The model organism names are abbreviated in the kinase names as follows: Hs, Hs, *Homo sapiens*; Dm, *Drosophila melanogaster*; Ce, *Caenorhabditis elegans*; Sc, *Saccharomyces cerevisiae*. The bootstrap values (> = 50) are showed at each node of the phylogenetic trees.
(TIF)

S7 Fig **Phylogram of the STE Kinases.** A phylogenetic tree based on the catalytic domains was constructed as described in the Methods section. Aminoglycoside kinase (APH3) was used as an outgroup. The microsporidian ePKs are labeled in red. The model organism names are abbreviated in the kinase name as follows: Hs, Hs, *Homo sapiens*; Dm, *Drosophila melanogaster*; Ce, *Caenorhabditis elegans*; Sc, *Saccharomyces cerevisiae*. The bootstrap values (> = 50) are showed at each node of the phylogenetic trees.
(TIF)

S1 Table **Draft Kinomes of the Microsporidia** ***Enterocytozoon bieneusi, Encephalitozoon cuniculi, Nosema bombycis*** **and** ***Nosema ceranae*** **.**
(XLSX)

## References

[1] Bhat SA, Bashir I, Kamili AS (2009) Microsporidiosis of silkworm, Bombyx mori L.(Lepidoptera-Bombycidae): a review. Afr J Agric Res 4:1519–1523.

[2] Chen Y, Evans JD, Smith IB, Pettis JS (2008) Nosema ceranae is a long-present and wide-spread microsporidian infection of the European honey bee (Apis mellifera) in the United States. Journal of Invertebrate Pathology 97:186–188. 17880997 10.1016/j.jip.2007.07.010

[3] Cox-Foster DL, Conlan S, Holmes EC, Palacios G, Evans JD, et al (2007) A metagenomic survey of microbes in honey bee colony collapse disorder. Science 318:283–287. 17823314 10.1126/science.1146498

[4] Higes M, Martín-Hernández R, Botías C, Bailón EG, González-Porto AV, et al (2008) How natural infection by Nosema ceranae causes honeybee colony collapse. Environmental Microbiology 10:2659–2669. 18647336 10.1111/j.1462-2920.2008.01687.x

[5] Capella-Gutiérrez S, Marcet-Houben M, Gabaldón T (2012) Phylogenomics supports microsporidia as the earliest diverging clade of sequenced fungi. BMC biology 10:47. 22651672 10.1186/1741-7007-10-47 PMC3586952

[6] Goldberg AV, Molik S, Tsaousis AD, Neumann K, Kuhnke G, et al (2008) Localization and functionality of microsporidian iron–sulphur cluster assembly proteins. Nature 452:624–628. 18311129 10.1038/nature06606

[7] Williams BA, Hirt RP, Lucocq JM, Embley TM (2002) A mitochondrial remnant in the microsporidian Trachipleistophora hominis. Nature 418:865–869. 12192407 10.1038/nature00949

[8] Katinka MD, Duprat S, Cornillot E, Méténier G, Thomarat F, et al (2001) Genome sequence and gene compaction of the eukaryote parasite Encephalitozoon cuniculi. Nature 414:450–453. 11719806 10.1038/35106579

[9] Wittner M, Weiss LM (1999) The microsporidia and microsporidiosis: ASM Press Washington, DC:.

[10] Xu Y, Weiss LM (2005) The microsporidian polar tube: a highly specialised invasion organelle. Int J Parasitol 35:941–953. 16005007 10.1016/j.ijpara.2005.04.003 PMC3109658

[11] Ward P, Equinet L, Packer J, Doerig C (2004) Protein kinases of the human malaria parasite Plasmodium falciparum: the kinome of a divergent eukaryote. BMC genomics 5:79. 15479470 10.1186/1471-2164-5-79 PMC526369

[12] Parsons M, Worthey E, Ward P, Mottram J (2005) Comparative analysis of the kinomes of three pathogenic trypanosomatids: Leishmania major, Trypanosoma brucei and Trypanosoma cruzi. BMC genomics 6:127. 16164760 10.1186/1471-2164-6-127 PMC1266030

[13] Hanks SK (2003) Genomic analysis of the eukaryotic protein kinase superfamily: a perspective. Genome Biol 4:111. 12734000 10.1186/gb-2003-4-5-111 PMC156577

[14] Miranda-Saavedra D, Barton GJ (2007) Classification and functional annotation of eukaryotic protein kinases. Proteins: Structure, Function, and Bioinformatics 68:893–914. 10.1002/prot.21444 17557329

[15] Hanks SK, Hunter T (1995) Protein kinases 6. The eukaryotic protein kinase superfamily: kinase (catalytic) domain structure and classification. The FASEB Journal 9:576–596. 7768349

[16] Manning G, Plowman GD, Hunter T, Sudarsanam S (2002) Evolution of protein kinase signaling from yeast to man. Trends in biochemical sciences 27:514–520. 12368087 10.1016/s0968-0004(02)02179-5

[17] Yang DL, Dang XQ, Tian R, Long MX, Li CF, et al (2014) Development of an approach to analyze the interaction between Nosema bombycis (microsporidia) deproteinated chitin spore coats and spore wall proteins. Journal of Invertebrate Pathology 115:1–7. 24161881 10.1016/j.jip.2013.10.004

[18] Scheeff ED, Axelrod HL, Miller MD, Chiu HJ, Deacon AM, et al (2010) Genomics, evolution, and crystal structure of a new family of bacterial spore kinases. Proteins: Structure, Function, and Bioinformatics 78:1470–1482. 10.1002/prot.22663 PMC2860764 20077512

[19] Miranda-Saavedra D, Stark MJ, Packer JC, Vivares CP, Doerig C, et al (2007) The complement of protein kinases of the microsporidium Encephalitozoon cuniculi in relation to those of Saccharomyces cerevisiae and Schizosaccharomyces pombe. BMC genomics 8:309. 17784954 10.1186/1471-2164-8-309 PMC2078597

[20] Pan G, Xu J, Li T, Xia Q, Liu S-L, et al (2013) Comparative genomics of parasitic silkworm microsporidia reveal an association between genome expansion and host adaptation. BMC genomics 14:186. 23496955 10.1186/1471-2164-14-186 PMC3614468

[21] Finn RD, Clements J, Eddy SR (2011) HMMER web server: interactive sequence similarity searching. Nucleic Acids Res 39:W29–37. 21593126 10.1093/nar/gkr367 PMC3125773

[22] Sonnhammer EL, Eddy SR, Birney E, Bateman A, Durbin R (1998) Pfam: multiple sequence alignments and HMM-profiles of protein domains. Nucleic acids research 26:320–322. 9399864 10.1093/nar/26.1.320 PMC147209

[23] Schultz J, Copley RR, Doerks T, Ponting CP, Bork P (2000) SMART: a web-based tool for the study of genetically mobile domains. Nucleic acids research 28:231–234. 10592234 10.1093/nar/28.1.231 PMC102444

[24] Shiu SH, Bleecker AB (2003) Expansion of the receptor-like kinase/Pelle gene family and receptor-like proteins in Arabidopsis. Plant Physiol 132:530–543. 12805585 10.1104/pp.103.021964 PMC166995

[25] Edgar RC (2004) MUSCLE: multiple sequence alignment with high accuracy and high throughput. Nucleic Acids Res 32:1792–1797. 15034147 10.1093/nar/gkh340 PMC390337

[26] Crooks GE, Hon G, Chandonia JM, Brenner SE (2004) WebLogo: a sequence logo generator. Genome Res 14:1188–1190. 15173120 10.1101/gr.849004 PMC419797

[27] Manning G, Whyte DB, Martinez R, Hunter T, Sudarsanam S (2002) The protein kinase complement of the human genome. Science 298:1912–1934. 12471243 10.1126/science.1075762

[28] Jung J, Yi G, Sukno SA, Thon MR (2010) PoGO: Prediction of Gene Ontology terms for fungal proteins. BMC Bioinformatics 11:215. 20429880 10.1186/1471-2105-11-215 PMC2882390

[29] Swofford DL (2003) {PAUP*. Phylogenetic analysis using parsimony (* and other methods). Version 4.}.

[30] Hon WC, McKay GA, Thompson PR, Sweet RM, Yang DS, et al (1997) Structure of an enzyme required for aminoglycoside antibiotic resistance reveals homology to eukaryotic protein kinases. Cell 89:887–895. 9200607 10.1016/s0092-8674(00)80274-3

[31] Librado P, Rozas J (2009) DnaSP v5: a software for comprehensive analysis of DNA polymorphism data. Bioinformatics 25:1451–1452. 19346325 10.1093/bioinformatics/btp187

[32] Manning G, Reiner DS, Lauwaet T, Dacre M, Smith A, et al (2011) The minimal kinome of Giardia lamblia illuminates early kinase evolution and unique parasite biology. Genome Biol 12:R66. 21787419 10.1186/gb-2011-12-7-r66 PMC3218828

[33] Kosti I, Mandel-Gutfreund Y, Glaser F, Horwitz BA (2010) Comparative analysis of fungal protein kinases and associated domains. BMC Genomics 11:133. 20178650 10.1186/1471-2164-11-133 PMC2838846

[34] Lower BH, Kennelly PJ (2003) Open reading frame sso2387 from the archaeon Sulfolobus solfataricus encodes a polypeptide with protein-serine kinase activity. J Bacteriol 185:3436–3445. 12754243 10.1128/JB.185.11.3436-3445.2003 PMC155377

[35] Bush EW, Helmke SM, Birnbaum RA, Perryman MB (2000) Myotonic dystrophy protein kinase domains mediate localization, oligomerization, novel catalytic activity, and autoinhibition. Biochemistry 39:8480–8490. 10913253 10.1021/bi992142f

[36] Masek KS, Fiore J, Leitges M, Yan SF, Freedman BD, et al (2006) Host cell Ca2+ and protein kinase C regulate innate recognition of Toxoplasma gondii. J Cell Sci 119:4565–4573. 17074836 10.1242/jcs.03206

[37] St-Denis A, Caouras V, Gervais F, Descoteaux A (1999) Role of protein kinase C-alpha in the control of infection by intracellular pathogens in macrophages. J Immunol 163:5505–5511. 10553077

[38] Riedel H, Parissenti AM, Hansen H, Su L, Shieh HL (1993) Stimulation of calcium uptake in Saccharomyces cerevisiae by bovine protein kinase C alpha. J Biol Chem 268:3456–3462. 8429022

[39] Hatanaka M, Shimoda C (2001) The cyclic AMP/PKA signal pathway is required for initiation of spore germination in Schizosaccharomyces pombe. Yeast 18:207–217. 11180454 10.1002/1097-0061(200102)18:3<207::AID-YEA661>3.0.CO;2-I

[40] Toda T, Cameron S, Sass P, Zoller M, Wigler M (1987) Three different genes in S. cerevisiae encode the catalytic subunits of the cAMP-dependent protein kinase. Cell 50:277–287. 3036373 10.1016/0092-8674(87)90223-6

[41] Inselburg J (1983) Stage-specific inhibitory effect of cyclic AMP on asexual maturation and gametocyte formation of Plasmodium falciparum. J Parasitol 69:592–597. 6313893

[42] Williams JG, Harwood AJ, Hopper NA, Simon MN, Bouzid S, et al (1993) Regulation of Dictyostelium morphogenesis by cAMP-dependent protein kinase. Philos Trans R Soc Lond B Biol Sci 340:305–313. 8103933 10.1098/rstb.1993.0072

[43] Vinals F, Chambard JC, Pouyssegur J (1999) p70 S6 kinase-mediated protein synthesis is a critical step for vascular endothelial cell proliferation. Journal of Biological Chemistry 274:26776–26782. 10480882 10.1074/jbc.274.38.26776

[44] Mylonakis E, Idnurm A, Moreno R, El Khoury J, Rottman JB, et al (2004) Cryptococcus neoformans Kin1 protein kinase homologue, identified through a Caenorhabditis elegans screen, promotes virulence in mammals. Mol Microbiol 54:407–419. 15469513 10.1111/j.1365-2958.2004.04310.x

[45] Lehmler C, Steinberg G, Snetselaar KM, Schliwa M, Kahmann R, et al (1997) Identification of a motor protein required for filamentous growth in Ustilago maydis. EMBO J 16:3464–3473. 9218789 10.1093/emboj/16.12.3464 PMC1169972

[46] Steinberg G, Schliwa M, Lehmler C, Bolker M, Kahmann R, et al (1998) Kinesin from the plant pathogenic fungus Ustilago maydis is involved in vacuole formation and cytoplasmic migration. J Cell Sci 111 (Pt 15):2235–2246. 10.1242/jcs.111.15.2235 9664045

[47] Drewes G, Nurse P (2003) The protein kinase kin1, the fission yeast orthologue of mammalian MARK/PAR-1, localises to new cell ends after mitosis and is important for bipolar growth. FEBS Lett 554:45–49. 14596912 10.1016/s0014-5793(03)01080-9

[48] Levin DE, Bishop JM (1990) A Putative Protein-Kinase Gene (Kin1+) Is Important for Growth Polarity in Schizosaccharomyces-Pombe. Proceedings of the National Academy of Sciences of the United States of America 87:8272–8276. 2236039 10.1073/pnas.87.21.8272 PMC54937

[49] Krylov DM, Nasmyth K, Koonin EV (2003) Evolution of eukaryotic cell cycle regulation: stepwise addition of regulatory kinases and late advent of the CDKs. Curr Biol 13:173–177. 12546794 10.1016/s0960-9822(03)00008-3

[50] Mielnichuk N, Sgarlata C, Perez-Martin J (2009) A role for the DNA-damage checkpoint kinase Chk1 in the virulence program of the fungus Ustilago maydis. J Cell Sci 122:4130–4140. 19861497 10.1242/jcs.052233

[51] Lev S, Sharon A, Hadar R, Ma H, Horwitz BA (1999) A mitogen-activated protein kinase of the corn leaf pathogen Cochliobolus heterostrophus is involved in conidiation, appressorium formation, and pathogenicity: diverse roles for mitogen-activated protein kinase homologs in foliar pathogens. Proc Natl Acad Sci U S A 96:13542–13547. 10557357 10.1073/pnas.96.23.13542 PMC23984

[52] Liao H, Byeon IJ, Tsai MD (1999) Structure and function of a new phosphopeptide-binding domain containing the FHA2 of Rad53. J Mol Biol 294:1041–1049. 10588905 10.1006/jmbi.1999.3313

[53] Pike BL, Yongkiettrakul S, Tsai MD, Heierhorst J (2003) Diverse but overlapping functions of the two forkhead-associated (FHA) domains in Rad53 checkpoint kinase activation. J Biol Chem 278:30421–30424. 12805372 10.1074/jbc.C300227200

[54] Schwartz MF, Lee SJ, Duong JK, Eminaga S, Stern DF (2003) FHA domain-mediated DNA checkpoint regulation of Rad53. Cell Cycle 2:384–396. 12851493

[55] Harmon AC, Gribskov M, Harper JF (2000) CDPKs - a kinase for every Ca2+ signal? Trends Plant Sci 5:154–159. 10740296 10.1016/s1360-1385(00)01577-6

[56] Billker O, Lourido S, Sibley LD (2009) Calcium-dependent signaling and kinases in apicomplexan parasites. Cell Host Microbe 5:612–622. 19527888 10.1016/j.chom.2009.05.017 PMC2718762

[57] Harper JF, Harmon A (2005) Plants, symbiosis and parasites: a calcium signalling connection. Nat Rev Mol Cell Biol 6:555–566. 16072038 10.1038/nrm1679

[58] Pines J (1995) Cyclins and Cyclin-Dependent Kinases - a Biochemical View. Biochemical Journal 308:697–711. 8948422 10.1042/bj3080697 PMC1136782

[59] Iino Y, Hiramine Y, Yamamoto M (1995) The role of cdc2 and other genes in meiosis in Schizosaccharomyces pombe. Genetics 140:1235–1245. 7498766 10.1093/genetics/140.4.1235 PMC1206690

[60] Carroll AS, O’Shea EK (2002) Pho85 and signaling environmental conditions. Trends Biochem Sci 27:87–93. 11852246 10.1016/s0968-0004(01)02040-0

[61] Harper JW, Elledge SJ (1998) The role of Cdk7 in CAK function, a retro-retrospective. Genes Dev 12:285–289. 9450924 10.1101/gad.12.3.285

[62] Larochelle S, Pandur J, Fisher RP, Salz HK, Suter B (1998) Cdk7 is essential for mitosis and for in vivo Cdk-activating kinase activity. Genes & development 12:370–381. 9450931 10.1101/gad.12.3.370 PMC316490

[63] Fisher RP (2005) Secrets of a double agent: CDK7 in cell-cycle control and transcription. Journal of cell science 118:5171–5180. 16280550 10.1242/jcs.02718

[64] Idanheimo N, Gauthier A, Salojarvi J, Siligato R, Brosche M, et al (2014) The Arabidopsis thaliana cysteine-rich receptor-like kinases CRK6 and CRK7 protect against apoplastic oxidative stress. Biochem Biophys Res Commun 445:457–462. 24530916 10.1016/j.bbrc.2014.02.013

[65] Ko TK, Kelly E, Pines J (2001) CrkRS: a novel conserved Cdc2-related protein kinase that colocalises with SC35 speckles. J Cell Sci 114:2591–2603. 11683387 10.1242/jcs.114.14.2591

[66] Aranda S, Laguna A, de la Luna S (2011) DYRK family of protein kinases: evolutionary relationships, biochemical properties, and functional roles. FASEB J 25:449–462. 21048044 10.1096/fj.10-165837

[67] Perrimon N, Smouse D (1989) Multiple functions of a Drosophila homeotic gene, zeste-white 3, during segmentation and neurogenesis. Dev Biol 135:287–305. 2570722 10.1016/0012-1606(89)90180-2

[68] Simpson P, Elmessal M, Delprado JM, Ripoll P (1988) Stripes of Positional Homologies across the Wing Blade of Drosophila-Melanogaster. Development 103:391–401.

[69] Harwood AJ, Plyte SE, Woodgett J, Strutt H, Kay RR (1995) Glycogen-Synthase Kinase-3 Regulates Cell Fate in Dictyostelium. Cell 80:139–148. 7813009 10.1016/0092-8674(95)90458-1

[70] Yost C, Torres M, Miller RR, Huang E, Kimelman D, et al (1996) The axis-inducing activity, stability, and subcellular distribution of beta-catenin is regulated in Xenopus embryos by glycogen synthase kinase 3. Genes & Development 10:1443–1454. 8666229 10.1101/gad.10.12.1443

[71] He X, Saintjeannet JP, Woodgett JR, Varmus HE, Dawid IB (1995) Glycogen-Synthase Kinase-3 and Dorsoventral Patterning in Xenopus Embryos (Vol 374, Pg 617, 1995). Nature 375:253–253. 10.1038/374617a0 7715701

[72] Coccetti P, Rossi RL, Sternieri F, Porro D, Russo GL, et al (2004) Mutations of the CK2 phosphorylation site of Sic1 affect cell size and S-Cdk kinase activity in Saccharomyces cerevisiae. Mol Microbiol 51:447–460. 14756785 10.1046/j.1365-2958.2003.03836.x

[73] Coccetti P, Zinzalla V, Tedeschi G, Russo GL, Fantinato S, et al (2006) Sic1 is phosphorylated by CK2 on Ser201 in budding yeast cells. Biochemical and Biophysical Research Communications 346:786–793. 16777072 10.1016/j.bbrc.2006.05.171

[74] Coccetti P, Tripodi F, Tedeschi G, Nonnis S, Marin O, et al (2008) The CK2 phosphorylation of catalytic domain of Cdc34 modulates its activity at the G1 to S transition in Saccharomyces cerevisiae. Cell Cycle 7:1391–1401. 18418079 10.4161/cc.7.10.5825

[75] Bidwai AP, Reed JC, Glover CV (1995) Cloning and disruption of CKB1, the gene encoding the 38-kDa beta subunit of Saccharomyces cerevisiae casein kinase II (CKII). Deletion of CKII regulatory subunits elicits a salt-sensitive phenotype. J Biol Chem 270:10395–10404. 7737972 10.1074/jbc.270.18.10395

[76] Noselli S (1998) JNK signaling and morphogenesis in Drosophila. Trends Genet 14:33–38. 9448464 10.1016/S0168-9525(97)01320-6

[77] Stronach B, Perrimon N (2002) Activation of the JNK pathway during dorsal closure in Drosophila requires the mixed lineage kinase, slipper. Genes Dev 16:377–387. 11825878 10.1101/gad.953002 PMC155330

[78] Zhang W, Liu HT (2002) MAPK signal pathways in the regulation of cell proliferation in mammalian cells. Cell Res 12:9–18. 11942415 10.1038/sj.cr.7290105

[79] Rangarajan R, Bei AK, Jethwaney D, Maldonado P, Dorin D, et al (2005) A mitogen-activated protein kinase regulates male gametogenesis and transmission of the malaria parasite Plasmodium berghei. EMBO Rep 6:464–469. 15864297 10.1038/sj.embor.7400404 PMC1299310

[80] Dohrmann PR, Sclafani RA (2006) Novel role for checkpoint Rad53 protein kinase in the initiation of chromosomal DNA replication in Saccharomyces cerevisiae. Genetics 174:87–99. 16816422 10.1534/genetics.106.060236 PMC1569810

[81] Plowman GD, Sudarsanam S, Bingham J, Whyte D, Hunter T (1999) The protein kinases of Caenorhabditis elegans: a model for signal transduction in multicellular organisms. Proc Natl Acad Sci U S A 96:13603–13610. 10570119 10.1073/pnas.96.24.13603 PMC24111

[82] Robert-Gangneux F, Creuzet C, Dupouy-Camet J, Roisin MP (2001) [Mitogen activated protein kinases (MAPK) and Toxoplasma gondii host cell invasion]. Ann Pharm Fr 59:297–304. 11787422

[83] Andrade LF, Nahum LA, Avelar LG, Silva LL, Zerlotini A, et al (2011) Eukaryotic protein kinases (ePKs) of the helminth parasite Schistosoma mansoni. BMC Genomics 12:215. 21548963 10.1186/1471-2164-12-215 PMC3117856

[84] Yoon HJ, Loo S, Campbell JL (1993) Regulation of Saccharomyces cerevisiae CDC7 function during the cell cycle. Mol Biol Cell 4:195–208. 8382976 10.1091/mbc.4.2.195 PMC300915

[85] Ohtoshi A, Miyake T, Arai K, Masai H (1997) Analyses of Saccharomyces cerevisiae Cdc7 kinase point mutants: dominant-negative inhibition of DNA replication on overexpression of kinase-negative Cdc7 proteins. Mol Gen Genet 254:562–570. 9197416 10.1007/s004380050452

[86] Carmena M, Earnshaw WC (2003) The cellular geography of aurora kinases. Nat Rev Mol Cell Biol 4:842–854. 14625535 10.1038/nrm1245

[87] Lee KS, Erikson RL (1997) Plk is a functional homolog of Saccharomyces cerevisiae Cdc5, and elevated Plk activity induces multiple septation structures. Molecular and Cellular Biology 17:3408–3417. 9154840 10.1128/mcb.17.6.3408 PMC232194

[88] Russell P, Nurse P (1987) Negative Regulation of Mitosis by Wee1+, a Gene Encoding a Protein-Kinase Homolog. Cell 49:559–567. 3032459 10.1016/0092-8674(87)90458-2

[89] Panigada D, Grianti P, Nespoli A, Rotondo G, Castro DG, et al (2013) Yeast Haspin Kinase Regulates Polarity Cues Necessary for Mitotic Spindle Positioning and Is Required to Tolerate Mitotic Arrest. Developmental Cell 26:483–495. 23973165 10.1016/j.devcel.2013.07.013

[90] Verissimo F, Jordan P (2001) WNK kinases, a novel protein kinase subfamily in multi-cellular organisms. Oncogene 20:5562–5569. 11571656 10.1038/sj.onc.1204726

[91] Southern TR, Jolly CE, Lester ME, Hayman JR (2007) EnP1, a microsporidian spore wall protein that enables spores to adhere to and infect host cells in vitro. Eukaryotic cell 6:1354–1362. 17557882 10.1128/EC.00113-07 PMC1951136

[92] Li Y, Wu Z, Pan G, He W, Zhang R, et al (2009) Identification of a novel spore wall protein (SWP26) from microsporidia *Nosema bombycis* . International journal for parasitology 39:391–398. 18854188 10.1016/j.ijpara.2008.08.011

[93] Takada R, Hijikata H, Kondoh H, Takada S (2005) Analysis of combinatorial effects of Wnts and Frizzleds on beta-catenin/armadillo stabilization and Dishevelled phosphorylation. Genes Cells 10:919–928. 16115200 10.1111/j.1365-2443.2005.00889.x

[94] Lee H, Chen RM, Lee Y, Yoo S, Lee C (2009) Essential roles of CKI delta and CKI epsilon in the mammalian circadian clock. Proceedings of the National Academy of Sciences of the United States of America 106:21359–21364. 19948962 10.1073/pnas.0906651106 PMC2795500

[95] Marchal C, Dupre S, Urban-Grimal D (2002) Casein kinase I controls a late step in the endocytic trafficking of yeast uracil permease. J Cell Sci 115:217–226. 11801739 10.1242/jcs.115.1.217

[96] Marchal C, Haguenauer-Tsapis R, Urban-Grimal D (2000) Casein kinase I-dependent phosphorylation within a PEST sequence and ubiquitination at nearby lysines signal endocytosis of yeast uracil permease. J Biol Chem 275:23608–23614. 10811641 10.1074/jbc.M001735200

[97] Hicke L, Zanolari B, Riezman H (1998) Cytoplasmic tail phosphorylation of the alpha-factor receptor is required for its ubiquitination and internalization. J Cell Biol 141:349–358. 9548714 10.1083/jcb.141.2.349 PMC2148449

[98] Feng Y, Davis NG (2000) Akr1p and the type I casein kinases act prior to the ubiquitination step of yeast endocytosis: Akr1p is required for kinase localization to the plasma membrane. Mol Cell Biol 20:5350–5359. 10866691 10.1128/mcb.20.14.5350-5359.2000 PMC85984

[99] Keeling PJ, Luker MA, Palmer JD (2000) Evidence from beta-tubulin phylogeny that microsporidia evolved from within the fungi. Molecular Biology and Evolution 17:23–31. 10666703 10.1093/oxfordjournals.molbev.a026235

[100] Morrison DK, Murakami MS, Cleghon V (2000) Protein kinases and phosphatases in the Drosophila genome. J Cell Biol 150:F57–62. 10908587 10.1083/jcb.150.2.f57 PMC2180215

[101] Qi M, Elion EA (2005) MAP kinase pathways. J Cell Sci 118:3569–3572. 16105880 10.1242/jcs.02470

[102] Miranda-Saavedra D, Stark MJ, Packer JC, Vivares CP, Doerig C, et al (2007) The complement of protein kinases of the microsporidium Encephalitozoon cuniculi in relation to those of Saccharomyces cerevisiae and Schizosaccharomyces pombe. BMC Genomics 8:309. 17784954 10.1186/1471-2164-8-309 PMC2078597

[103] Angermayr M, Roidl A, Bandlow W (2002) Yeast Rio1p is the founding member of a novel subfamily of protein serine kinases involved in the control of cell cycle progression. Molecular Microbiology 44:309–324. 11972772 10.1046/j.1365-2958.2002.02881.x

[104] Vanrobays E, Gleizes PE, Bousquet-Antonelli C, Noaillac-Depeyre J, Caizergues-Ferrer M, et al (2001) Processing of 20S pre-rRNA to 18S ribosomal RNA in yeast requires Rrp10p, an essential non-ribosomal cytoplasmic protein. Embo Journal 20:4204–4213. 11483523 10.1093/emboj/20.15.4204 PMC149176

[105] Xu Y, Takvorian P, Cali A, Wang F, Zhang H, et al (2006) Identification of a new spore wall protein from Encephalitozoon cuniculi. Infection and immunity 74:239–247. 16368977 10.1128/IAI.74.1.239-247.2006 PMC1346661

[106] Li Z, Pan G, Li T, Huang W, Chen J, et al (2012) SWP5, a spore wall protein, interacts with polar tube proteins in the parasitic microsporidian Nosema bombycis. Eukaryot Cell 11:229–237. 22140229 10.1128/EC.05127-11 PMC3272902

[107] Manning G (2005) Genomic overview of protein kinases. WormBook: 1–19. 10.1895/wormbook.1.60.1 PMC4780929 18050405

[108] Manning G, Plowman GD, Hunter T, Sudarsanam S (2002) Evolution of protein kinase signaling from yeast to man. Trends Biochem Sci 27:514–520. 12368087 10.1016/s0968-0004(02)02179-5

[109] Hergovich A, Stegert MR, Schmitz D, Hemmings BA (2006) NDR kinases regulate essential cell processes from yeast to humans. Nat Rev Mol Cell Biol 7:253–264. 16607288 10.1038/nrm1891

[110] Du K, Tsichlis PN (2005) Regulation of the Akt kinase by interacting proteins. Oncogene 24:7401–7409. 16288287 10.1038/sj.onc.1209099

