# Supplementary figures and images for: Genome-Wide Identification and Comprehensive Analyses of the Kinomes in Four Pathogenic Microsporidia Species
Source: PLoS One. 2014 Dec 30;9(12):e115890. doi: 10.1371/journal.pone.0115890 (PMC4280135; doi:10.1371/journal.pone.0115890)

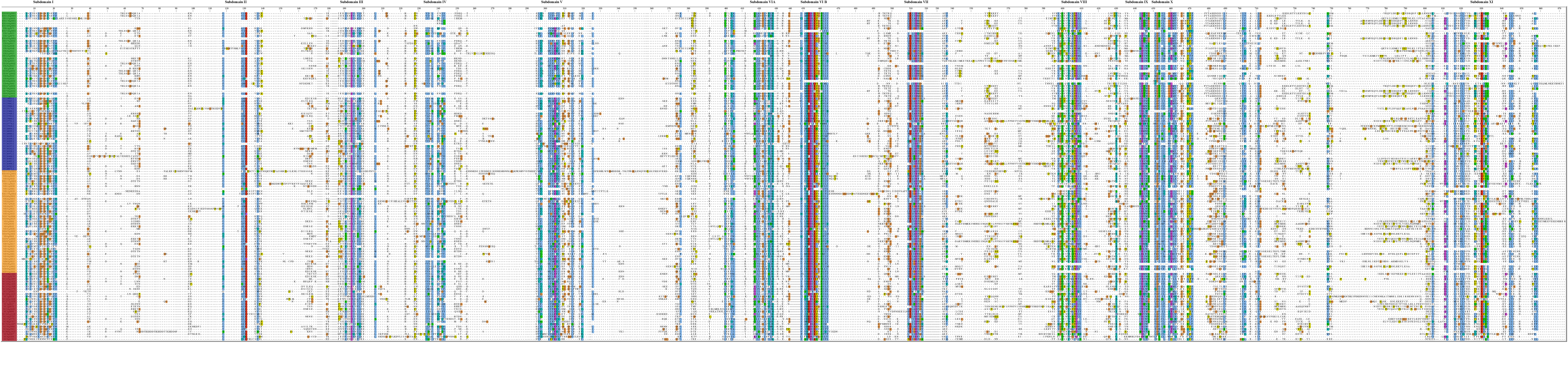

Supplement: S1 Fig — Multiple Sequence Alignment of the ePK Domains. The domain sequences were aligned without modifications. The kinase names are shaded according to species: green, Enterocytozoon bieneusi; purple, Encephalitozoon cuniculi; orange, Nosema bombycis; red, Nosema ceranae. The 12 conserved subdomains are numbered according to the Hanks and Hunter classification system. The positions of the conserved amino acid residues are indicated with Arabic numerals. (TIF) [file pone.0115890.s001.tif]
